# The Expression and Molecular Roles of MAMDC2 in MSS Colorectal Cancer with a High Tumor Stromal Ratio

**DOI:** 10.3390/biomedicines13051217

**Published:** 2025-05-17

**Authors:** Yiling Liu, Shengnan Qian, Jia Wei, Jianting He, Minghui Li, Xiaobing Gao, Hong Cai, Yiqing Wang, Yue Han, Tianyuan Tan, Minhui Yang

**Affiliations:** 1 Department of Pathology, School of Basic Medical Sciences, Southern Medical University, Guangzhou 510515, China; 2 Department of Pathology, Beijing Youan Hospital, Capital Medical University, Beijing 100069, China; 3 Department of Pathology, Nanfang Hospital, Southern Medical University, Guangzhou 510515, China

**Keywords:** MAM domain, colorectal cancer, consensus molecular subtypes (CMSs), epithelial–mesenchymal transition (EMT), cancer-associated fibroblasts (CAFs)

## Abstract

**Background:** Colorectal cancer (CRC) heterogeneity is strongly influenced by molecular subtypes and tumor stroma interactions. The meprin/A5/PTPmu (MAM) domain, a conserved structural motif in transmembrane proteins, remains undercharacterized in CRC pathogenesis. **Methods:** We analyzed RNA-seq data from TCGA-COAD to evaluate MAM domain gene expression. Immunohistochemistry and Western blotting were conducted to validate the results of the database analysis. **Results:** Bioinformatics analysis revealed that MAM domain-containing protein 2 (MAMDC2) was enriched in mesenchymal subtype 4 (CMS4) colorectal cancer (*p* < 0.001). IHC confirmed MAMDC2 overexpression in MSS colorectal cancer with a high tumor stroma ratio (TSR) and peritoneal metastatic lesions (*p* < 0.01). WB and real-time PCR analyses confirmed that MAMDC2 has a role in regulating epithelial–mesenchymal transition (EMT) development in CRC. Importantly, we identified that cancer cell-derived MAMDC2 promotes MYLK expression in cancer-associated fibroblasts (CAFs) through paracrine signaling. **Conclusions:** Our findings suggest MAMDC2 may function as a stromal-associated regulator in MSS colorectal cancer with a high tumor stromal ratio (TSR).

## 1. Introduction

Colorectal cancer (CRC) is a leading cause of cancer-related mortality worldwide. It is characterized by significant molecular and clinical heterogeneity [1]. The four consensus molecular subtypes (CMSs) are as follows: CMS1, CMS2, CMS3, and CMS4. CMS4 is associated with the poorest prognosis due to its highly desmoplastic tumor microenvironment, activation of TGF-β and epithelial–mesenchymal transition (EMT) pathways, and immune exclusion [2]. CMS4 colorectal cancer is enriched with cancer-associated fibroblasts (CAFs) and exhibits resistance to conventional therapies, including immunotherapy [2,3,4]. The intrinsic heterogeneity of CMS4, particularly in cases with peritoneal metastases (CMS4-PM), further complicates treatment strategies. Despite advances in targeting CAFs or TGF-β signaling, therapeutic outcomes remain suboptimal, highlighting the urgent need to elucidate the molecular mechanisms driving CMS4 progression and to identify novel therapeutic targets [5,6].

CAFs are a pivotal stromal constituent that critically modulate tumor progression, metastasis, metabolism, and therapeutic resistance through their dynamic regulation of tumor cell immune microenvironment interactions [7,8,9]. Notably, approximately one-third of CRCs display a phenotype enriched with CAFs. This phenotype is characterized by the secretion of key cytokines (e.g., TGF-β, IL-6) and extensive extracellular matrix remodeling [10]. These CAF-driven mechanisms collectively orchestrate an immunosuppressive and tumor-promoting microenvironment, fostering malignant progression and therapeutic evasion. However, the heterogeneity of CAFs and the complexity of their regulatory networks pose significant challenges for targeted therapies. Recent efforts to modulate the extracellular matrix (ECM), such as targeting collagen and hyaluronan, have shown promise in sensitizing CMS4 tumors to chemotherapy and immunotherapy [11,12,13]. It is clear that stromal/tumor crosstalk plays a critical role in the development of CRC. This emphasizes the need to discover new stromal-targeted therapeutic molecules.

MAM (meprin/A5/PTPmu) domain, a conserved structural motif present in numerous adhesion proteins, is essential for cellular communication and the dynamic interplay between cells and their surrounding extracellular environment [14]. In cancer, MAM domain-containing proteins, such as ALK and EGFL6, have been implicated in tumor progression and metastasis [15,16,17,18,19]. MAM domain-containing 2 (MAMDC2), also known as MAM domain-containing proteoglycan, spans 3085 base pairs, encodes a protein of 686 amino acids, and is predicted to initiate glycosaminoglycan-binding activity, functioning through chondroitin 4-sulfate glycosaminoglycan in the upstream or within peptide cross-linking [20].

MAMDC2 is expressed in many tissues and cell types, showing that it has many functions in different physiological and pathological situations. Recent investigations have revealed that MAMDC2 is expressed in adult skeletal muscle and differentiated myocytes, localized to the sarcoplasm and myonuclei. It is secreted by myoblasts and incorporated into myotubes within the extracellular compartment [21]. In Alzheimer’s disease, MAMDC2 is highly expressed in microglia and enhances STING-mediated type I interferon activation through its interaction with the TBK1-IRF3 signaling pathway [22]. In cancer, MAMDC2 has been definitively identified as a potential tumor suppressor in ER+ breast tumors, where its downregulation promotes tumor progression by attenuating MAPK signaling. Intriguingly, this tumor-suppressive function appears to be context-dependent, as MAMDC2 exhibits no significant regulatory effects in the more aggressive triple-negative breast cancer [23]. However, its role in CRC, particularly in the aggressive CMS4, remains unexplored.

In this study, we systematically analyzed the expression characteristics of MAM domain-containing genes in CRC and investigated the potential functional roles of MAMDC2 in MSS colorectal cancer with a high tumor stroma ratio (TSR). Our findings identify MAMDC2 as a stromal-associated effector of EMT–stromal crosstalk in MSS colorectal cancer with a high TSR, demonstrating its high expression in tumors with a high TSR and peritoneal metastases. Bioinformatics analyses of TCGA data and functional experiments revealed that MAMDC2 expression positively correlates with EMT-related markers (*CDH2*, *SNAI1*, and *MMP2*) and is associated with increased expression of these markers. Furthermore, we found that MAMDC2 upregulates MYLK expression in CAFs, suggesting its potential involvement in CAF activation. Our findings provide insights into the tumor stroma crosstalk mediated by MAMDC2 and may offer potential therapeutic targets for CMS4 CRC.

## 2. Materials and Methods

### 2.1. InterPro Dataset Analysis

The InterPro database (http://www.ebi.ac.uk/interpro/, accessed on 23 April 2023), provides functional analysis of proteins by classifying them into families and predicting domains and important sites [24]. Through systematic screening of the InterPro database, we identified all human genes encoding proteins that contain MAM domains.

### 2.2. Data Collection and Processing

RNA sequencing data and associated clinical metadata such as age, sex, tumor grade, survival status, and TNM staging were obtained from The Cancer Genome Atlas (TCGA, https://cancergenome.nih.gov, accessed on 4 June 2023). In this study, 480 CRC samples and 41 normal controls were included in the analysis. Differential gene expression analysis was performed using the DESeq2 package in R (4.2.1). We used ggplot2 to visualize the results. In addition, the CMScaller package was used to classify the TCGA-COAD dataset into consensus molecular subtypes (CMSs), allowing the analysis of expression profiles for all identified differentially expressed genes (DEGs) across different CMS categories.

Single-cell RNA sequencing datasets (accession numbers GSE183916 and GSE245552) were obtained from the Gene Expression Omnibus (GEO) database using the GEOquery R package. Raw gene expression matrices were processed and converted into Seurat objects for subsequent computational analyses. Stringent quality control criteria were applied to ensure data integrity, retaining only genes detected in a minimum of three cells and cells expressing at least 200 genes. Mitochondrial RNA content was quantified using the PercentageFeatureSet function, and cells exhibiting mitochondrial read proportions exceeding 25% were excluded from further analysis. Gene expression data were normalized using the NormalizeData function to mitigate technical variability and improve the accuracy of downstream analyses. Principal component analysis (PCA) was performed to reduce data dimensionality and identify dominant sources of variation for subsequent analytical steps. To address potential batch effects, we employed the Harmony algorithm via the RunHarmony function, integrating datasets based on PCA-derived embeddings. Cell clusters were annotated by cross-referencing with the CellMarker database, followed by manual validation using established marker genes. Cell-type-specific expression patterns of MAMDC2 were visualized using dot plots to assess its distribution across identified populations.

### 2.3. Kaplan–Meier Plotter

The Kaplan–Meier Plotter (https://kmplot.com/analysis/, accessed on 6 November 2024), an online platform designed to evaluate associations between gene expression (mRNA, miRNA, protein) and patient survival outcomes, was used in this study to investigate the prognostic significance of genes containing MAM domains in CRC. Specifically, this tool was used to predict overall survival (OS) based on the expression profiles of these genes.

### 2.4. Gene-Set Enrichment Analysis

Gene Ontology (GO, https://www.geneontology.org/, accessed on 14 April 2024) provides structured and computable insights into the functions of genes and their products, whereas the Kyoto Encyclopedia of Genes and Genomes (KEGG, http://www.kegg.jp/, accessed on 14 April 2024) serves as a comprehensive repository of gene functions and biological pathways [25,26]. In this study, we performed a gene enrichment analysis to explore the potential signaling pathways and biological functions associated with *MAMDC2*.

### 2.5. TIMER Analysis

The association between *MAMDC2* expression and immune/stromal cell infiltration was analyzed using the TIMER2.0 database (http://timer.cistrome.org/, accessed on 29 April 2024) [27]. Within the “Immune” module, *MAMDC2* was specified in the gene expression field, and the XCELL algorithm was applied to evaluate immune and stromal cells, with the “purity-adjusted” option enabled. Spearman correlation analysis was performed by default, where rho > 0 indicated a positive correlation, rho < 0 indicated a negative correlation, and *p* < 0.05 was considered statistically significant.

### 2.6. Immunohistochemistry

The inclusion criteria for this study were as follows: patients who underwent radical surgical resection with postoperative pathological confirmation of primary colonic or rectal adenocarcinoma (adenocarcinoma, not otherwise specified), with synchronous metastatic lesions in lymph nodes, liver, peritoneum, or omentum confirmed with pathology, and microsatellite stable stability (MSS) identified using molecular genetic testing. Exclusion criteria were as follows: patients with concomitant malignancies in other organs or multiple primary colorectal carcinomas, those who received neoadjuvant therapy prior to radical surgery, non-adenocarcinoma histological types (such as squamous cell carcinoma, neuroendocrine carcinoma, or lymphoma), tissue specimens with extensive necrosis affecting immunohistochemical evaluation, and cases with only small biopsy specimens that were insufficient for immunohistochemical analysis.

Immunohistochemistry (IHC) was used to evaluate the expression of MAMDC2 and MYLK. Tissue sections were initially deparaffinized in an oven, followed by deparaffinization in xylene and rehydration through a graded ethanol series. Antigen retrieval was performed by immersing in EDTA buffer (pH = 8.0; C1033, Solarbio, Beijing, China) and heating in boiling water for 5 min. Endogenous peroxidase activity was blocked with 3% hydrogen peroxide (H_2_O_2_), and nonspecific binding sites were blocked with 3% bovine serum albumin (BSA; IB9024, Solarbio, China) for 30 min at room temperature. Sections were then incubated overnight at 4 °C with a primary antibody (MAMDC2, ab121697, 1:100, Abcam, Shanghai, China; MYLK, 21642-1-AP, 1:400, ProteinTech Group, Wuhan, China), followed by incubation with a species-specific secondary antibody (PV-6000D, ZSGB-Bio, Beijing, China) for 1 h at room temperature. Target protein visualization was achieved using 3,3′-diaminobenzidine (DAB; PV-6000D, ZSGB-Bio, China) as a chromogen with a development time of 3–5 min and counterstaining with hematoxylin for 1–2 min. Finally, the sections were dehydrated through a graded ethanol series, cleared in xylene, and mounted in neutral balsam for microscopic examination.

Protein expression was assessed based on staining intensity and percentage of positive tumor cells: staining intensity: 0 (negative), 1 (weak, light yellow), 2 (moderate, brownish yellow), 3 (strong, dark brown); staining area (% positive cells): 0 (<5%), 1 (10–25%), 2 (25–50%), 3 (51–75%), 4 (76–100%). The final IHC score was calculated as (intensity score) × (area score). Cases with a total score > 4 were classified as high expression, while those with a total score ≤ 4 were considered low expression. All slides were independently evaluated by experienced pathologists blinded to clinical data.

### 2.7. Western Blotting

Briefly, equal amounts of protein were separated by SDS-PAGE and transferred to PVDF membranes (IPVH00010, Merck Millipore, Darmstadt, Germany). The membranes were blocked with NcmBlot blocking buffer (P30500, New Cell & Molecular Biotech, Suzhou, China) for 15 min at room temperature, followed by overnight incubation with primary antibodies (MAMDC2, ab121697, 1:1000, Abcam, China; MYLK, 21642-1-AP, 1:500, ProteinTech Group, China) at 4 °C. The membranes were then treated with peroxidase-conjugated secondary antibodies for 1 h at room temperature. Protein detection was performed with NcmECL UItra (P10200, New Cell & Molecular Biotech, Suzhou, China). The chemiluminescence signals was detected using the Tanon 5200 Multi image-analysis system (Tanon, China).

### 2.8. Cell Culture

All human CRC cell lines were obtained from the American Type Culture Collection (ATCC). CAF was obtained from the BeNa Culture Collection (BNCC). All CRC cell lines were cultured in RPMI 1640 medium containing 10% fetal bovine serum (FBS) at 37 °C under 5% CO_2_. CAF was cultured in DMEM containing 10% fetal bovine serum (FBS).

### 2.9. Plasmids and siRNAs

The plasmids expressing the full length of MAMDC2 were constructed by MiaoLingPlasmid (Wuhan, China). For siRNA treatment, siMAMDC2-1 (CCCAAUGUGAACUGGUUUGUU) and siMAMDC2-2 (UCCGUUUGGUCUACCAGAUAA) were synthesized by Hanyi Biosciences (Beijing, China). Plasmid and siRNA transfections were performed using jetPRIME transfection reagent (101000046, Polyplus Transfection, Illirch, France) following the manufacturer’s protocol.

### 2.10. Statistical Analysis

Data visualization was performed using GraphPad Prism v9.5.1 (GraphPad Software Inc., San Diego, CA, USA), and statistical significance between groups was assessed via Student’s *t*-test. Differences at *p* < 0.05 were considered statistically significant.

## 3. Results

### 3.1. Result 1: Differential Expression and Survival Analysis of MAM Domain-Containing Genes in Colorectal Cancer

**Figure 1:**
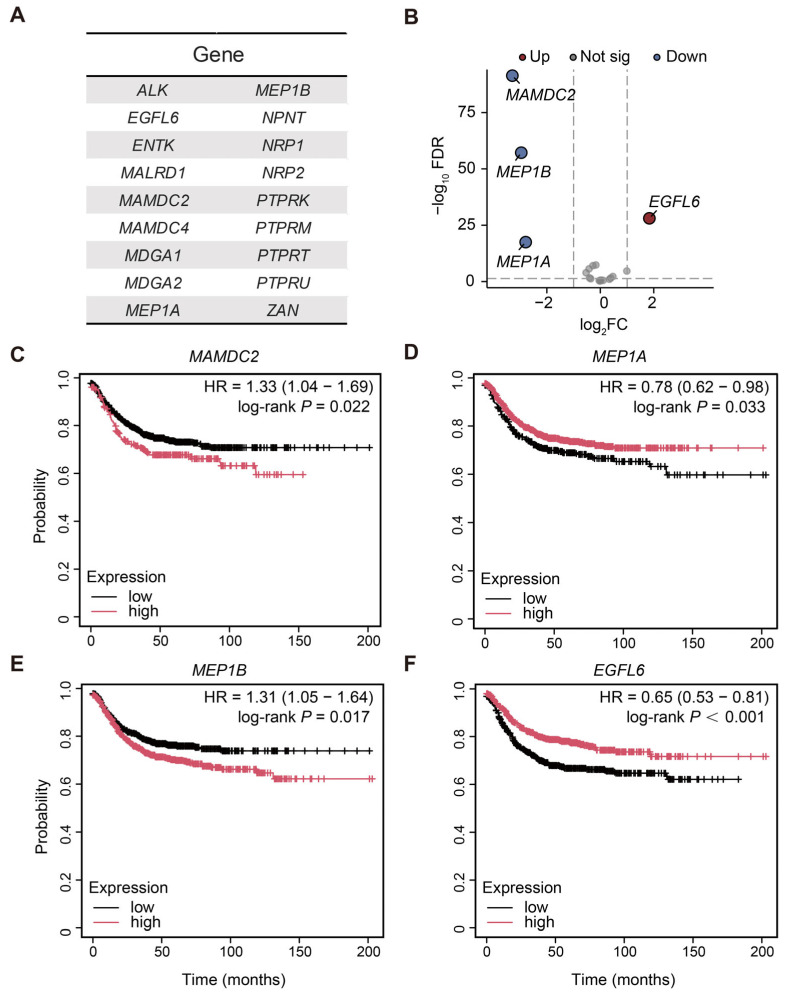
Differential expression and survival analysis of MAM domain-containing genes in colorectal cancer. (**A**) Identification of 18 MAM domain-containing genes via the InterPro database. (**B**) Volcano plot of differential mRNA expression of MAM domain-containing genes between normal and CRC tissues. (**C**–**F**) The Kaplan–Meier Plotter was used to determine the correlation between the mRNA levels of *MAMDC2* (**C**), *MEP1A* (**D**), *MEP1B* (**E**), and *EGFL6* (**F**) and the survival of CRC patients. *p* < 0.05.

Using the InterPro online database, we identified 18 human genes encoding proteins with MAM domains. These genes include *ALK*, *EGFL6*, *ENTK*, *MALRD1*, *MAMDC2*, *MAMDC4*, *MDGA1*, *MDGA2*, *MEP1A*, *MEP1B*, *NPNT*, *NRP1*, *NRP2*, *PTPRK*, *PTPRM*, *PTPRT*, *PTPRU*, and *ZAN* (Figure 1A).

The mRNA expression data for 18 genes from normal and CRC tissues were subsequently obtained from the TCGA-COAD database. As depicted in the volcano plot, differential expression analysis (|logFC| > 1 and FDR < 0.05) revealed a significant decrease in the expression levels of genes such as *MAMDC2*, *MEP1B*, and *MEP1A*, whereas *EGFL6* expression levels were higher in CRC tissues than in normal tissues (Figure 1B). The aforementioned genes, which contain the MAM domain, exhibit abnormal expression changes in CRC tissues, suggesting their potential involvement in tumorigenesis. Then we assessed the associations between CRC-specific differentially expressed MAM domain-containing genes (*MAMDC2*, *MEP1A*, *MEP1B*, and *EGFL6*) and the survival prognosis of patients with CRC. Kaplan–Meier analysis indicated that high expression levels of *MAMDC2* and *MEP1B* were associated with poorer overall survival (*MAMDC2*: HR = 1.33, 95% CI: 1.04–1.69, log-rank *p* = 0.022; *MEP1B*: HR = 1.31, 95% CI: 1.05–1.64, log-rank *p* = 0.017) (Figure 1C,E), suggesting that these genes might promote the development of CRC. Conversely, high expression levels of *MEP1A* and *EGFL6* were associated with better overall survival (*MEP1A*: hazard ratio (HR) = 0.78, 95% CI: 0.62–0.98, log-rank *p* = 0.033; *EGFL6*: HR = 0.65, 95% CI: 0.53–0.81, log-rank *p* < 0.001), suggesting their potential tumor suppressor roles.

### 3.2. Result 2: Differential Expression of MAM Domain-Containing Genes and Prognostic Association of MAMDC2 in CMSs

**Figure 2:**
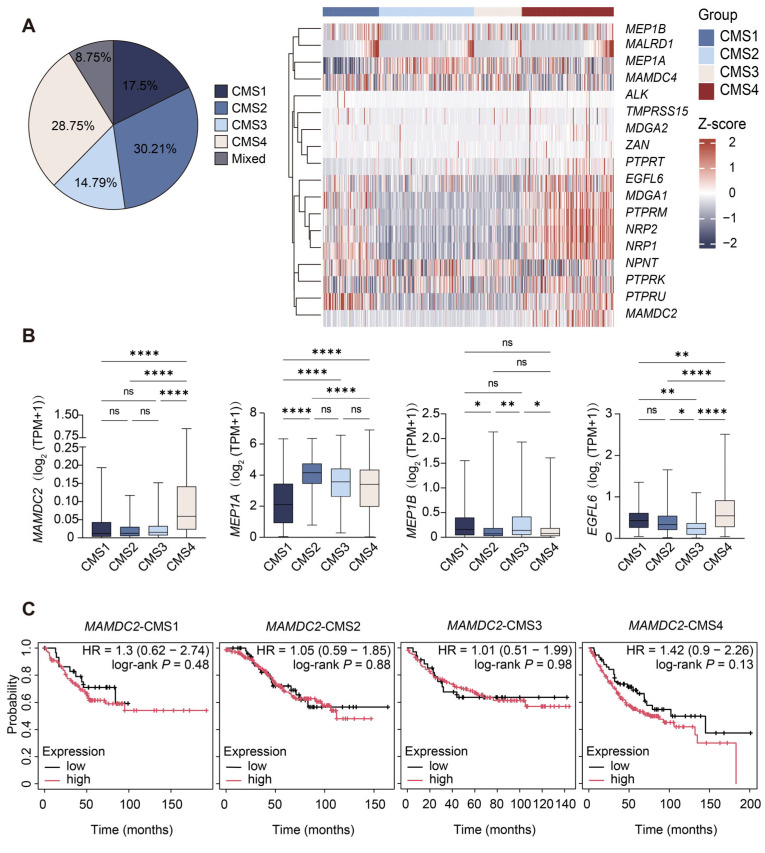
Differential expression of MAM domain-containing genes and prognostic association of MAMDC2 in CMSs. (**A**) CMS classification proportion of tumor samples in the TCGA COAD database (**left**) and differential expression of MAM domain-containing genes in CMS subtypes (**right**). n = 480. (**B**) Boxplots showing the differential expression of *MAMDC2*, *MEP1A*, *MEP1B*, and *EGFL6* in CMSs of CRC. (n (CMS1) = 84. n (CMS2) = 145. n (CMS3) = 71. n (CMS4) = 138). (**C**) Kaplan–Meier survival analysis of CMS1, CMS2, CMS3, and CMS4 patients stratified by *MAMDC2* expression. * *p* < 0.05, ** *p* < 0.01, **** *p* < 0.0001, ns = not significant.

We classified 480 tumor samples from the TCGA-COAD dataset into these subtypes, resulting in CMS1 (n = 84, 17.5%), CMS2 (n = 145, 30.2%), CMS3 (n = 71, 14.8%), CMS4 (n = 138, 28.8%), and mixed subtypes (n = 42, 8.75%), and focused on the differential expression of 18 MAM family genes in these CMS subtypes. *MAMDC2*, *NPR2*, and *PTPRM* were significantly upregulated in CMS4 compared to CMS1, CMS2, and CMS3. *PTPRU*, *MDGA1*, and *NRP1* were expressed at higher levels in both CMS1 and CMS4 than in CMS1 and CMS3 (Figure 2A). The boxplots illustrate the differential expression of MAM domain-containing genes between normal and tumor tissues within the CMS subtypes. *MAMDC2* showed significantly higher expression in CMS4 than in CMS1, CMS2, and CMS3 (*p* < 0.0001). *MEP1A* was expressed at low levels in CMS1, with significant differences compared with CMS2, CMS3, and CMS4 (*p* < 0.0001). In contrast, *MEP1B* exhibited no significant variation across subtypes. *EGFL6* expression was significantly lower in CMS3 than in the other subtypes (*p* < 0.05) (Figure 2B).

Given the aggressive nature of CMS4 and the marked upregulation of *MAMDC2* in this subtype, we further investigated whether *MAMDC2* expression levels could stratify survival outcomes specifically within CMS4. Kaplan–Meier analysis showed that CMS4 patients with high *MAMDC2* expression tended to have shorter overall survival compared to those with low expression (HR = 1.42, 95% CI: 0.9–2.26; log-rank *p* = 0.13). While this association did not reach statistical significance, *MAMDC2* demonstrated the highest HR among all CMS subgroups, indicating a potential trend toward poorer outcomes in this aggressive subtype. And this trend was specific to CMS4, as *MAMDC2* showed minimal prognostic impact in CMS1-3 subgroups (HR range: 1.01–1.3; log-rank *p* > 0.05). These findings suggest that *MAMDC2* may play a prominent role in CMS4 CRC (Figure 2C).

### 3.3. Result 3: Elevated MAMDC2 Expression in High-TSR Colorectal Cancer

**Figure 3:**
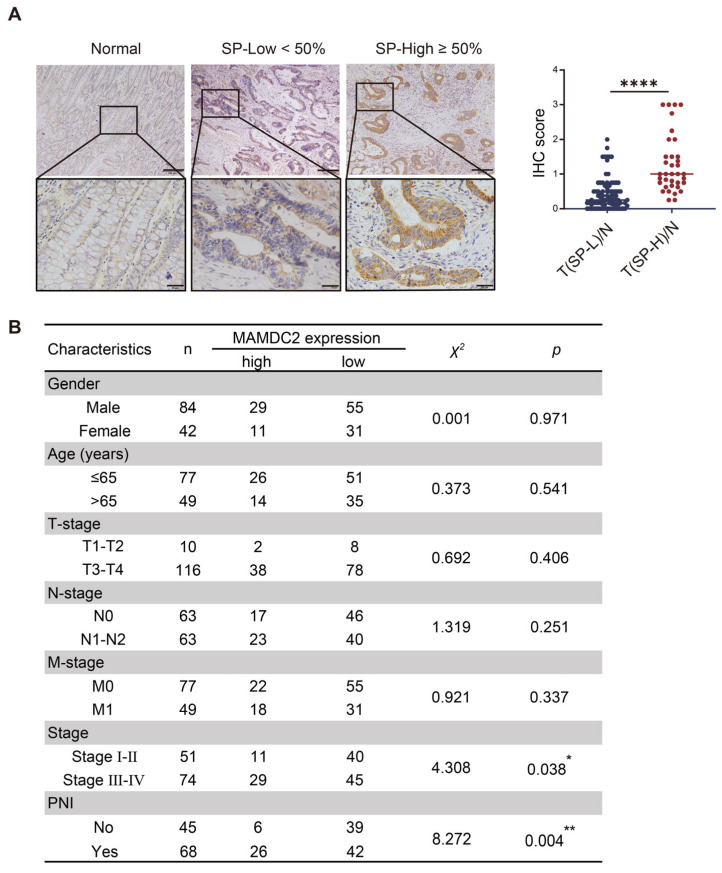
Elevated MAMDC2 expression in high-TSR colorectal cancer. (**A**) Representative images (**left**) and scores (**right**) of immunohistochemical analysis of MAMDC2 expression in CRC with different TSRs and in paired normal tissues. Original magnifications 40× and 100×. n (SP-L) = 85. n (SP-H) = 36. (**B**) Correlation between the clinicopathological features and expression of MAMDC2 in CRC. * *p* < 0.05, ** *p* < 0.01, **** *p* < 0.0001.

To further explore the expression level and localization of MAMDC2 in CRC tissues, we used immunohistochemistry (IHC) to test 121 paired CRC and normal tissue samples. These samples included 36 cases of high-TSR CRC and 85 cases of low-TSR CRC. The results revealed that MAMDC2 expression was significantly higher in high-TSR CRC than in low-TSR CRC (*p* < 0.0001), suggesting that the aberrant overexpression of MAMDC2 is closely related to the development of high-TSR CRC (Figure 3A). Notably, although the sample sizes were unbalanced between the two groups, post hoc power analysis using GPower_3.1.9.7 indicated a statistical power of 0.84 at α = 0.05 for detecting an effect size (Cohen’s d) of 0.59, supporting the reliability of this finding. To explore the clinical significance of MAMDC2 overexpression in high-TSR CRC, we analyzed its associations with clinicopathological parameters in the 121 paired samples. Patients were stratified into high and low MAMDC2 expression groups. Pearson chi-square tests revealed that MAMDC2 expression was significantly associated with advanced stage (*p* = 0.038) and perineural invasion (PNI; *p* = 0.004) but not with age or gender (Figure 3B). Meanwhile, univariate Cox regression analysis revealed a trend toward poorer prognosis in patients with high MAMDC2 expression (HR = 2.240, 95% CI: 0.603–8.315, *p* = 0.228), although this association did not reach statistical significance. These findings suggest that elevated MAMDC2 expression may contribute to the progression and aggressiveness of high-TSR CRC.

### 3.4. Result 4: MAMDC2 Expression Patterns in Primary and Metastatic Colorectal Cancer

**Figure 4:**
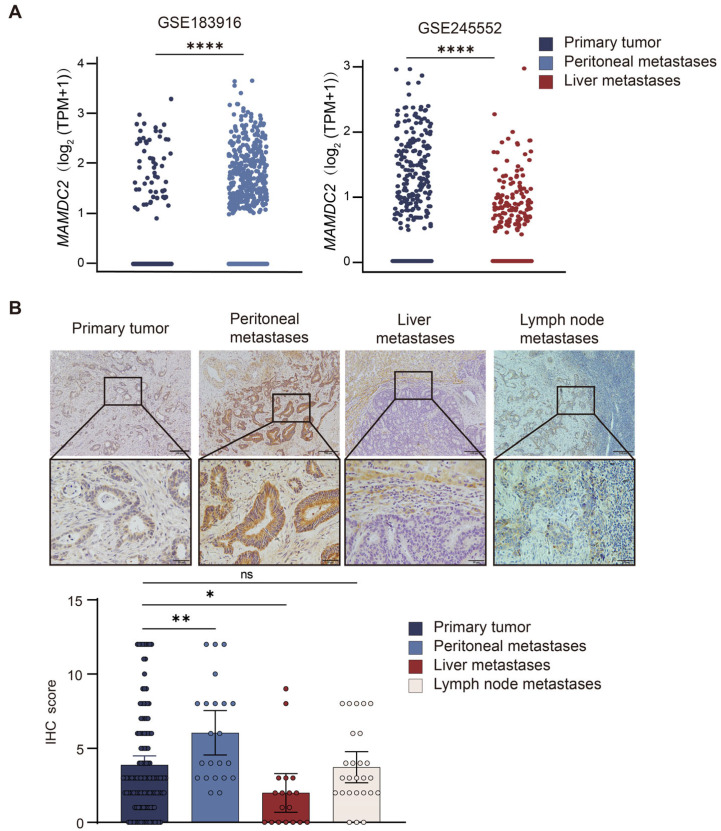
MAMDC2 expression patterns in primary and metastatic colorectal cancer. (**A**) *MAMDC2* expression in peritoneal and liver metastases from the GSE183916 and GSE245552 datasets. (**B**) Representative images (**above**) and scores (**below**) of immunohistochemical analysis of the expression of MAMDC2 in primary and metastatic lesions of colorectal cancer. Original magnifications 40× and 100×. n (peritoneal metastases) = 22. n (liver metastases) = 18. n (lymph node metastases) = 26. * *p* < 0.05, ** *p* < 0.01, **** *p* < 0.0001, ns = not significant.

To clarify the correlation between *MAMDC2* and CRC metastasis, we analyzed the expression of *MAMDC2* in peritoneal and liver metastases in the GSE183916 and GSE245552 datasets. The results showed that *MAMDC2* expression was decreased in liver metastases and increased in peritoneal metastases (*p* < 0.0001) (Figure 4A). Some studies have shown that the occurrence of liver metastases (LM) in CRC is often accompanied by a more frequent switch in CMS classification, of which CMS2 accounts for approximately 60%, while in peritoneal metastases (PM), the switch in subtype is relatively simple, mostly into CMS4 [28]. These findings suggest that MAMDC2 expression is positively correlated with increased stromal infiltration. Therefore, we performed IHC analysis on samples from patients with concurrent distant metastases, including 18 samples from patients with LM, 26 samples from patients with lymph node metastases (LN), and 22 samples from patients with PM. Statistical analysis revealed that, compared with that in primary tumors, the protein expression level of MAMDC2 was significantly higher in PM (*p* < 0.01), while it was significantly lower in LM (*p* < 0.05), and there was no significant difference in LN (*p* = 0.824) (Figure 4B). These results suggest that MAMDC2 is more likely to be highly expressed in CRC with abundant stroma, indicating that MAMDC2 may play a key role in the microenvironment of CRC with abundant stroma.

### 3.5. Result 5: MAMDC2 Regulates Epithelial–Mesenchymal Transition in Colorectal Cancer

**Figure 5:**
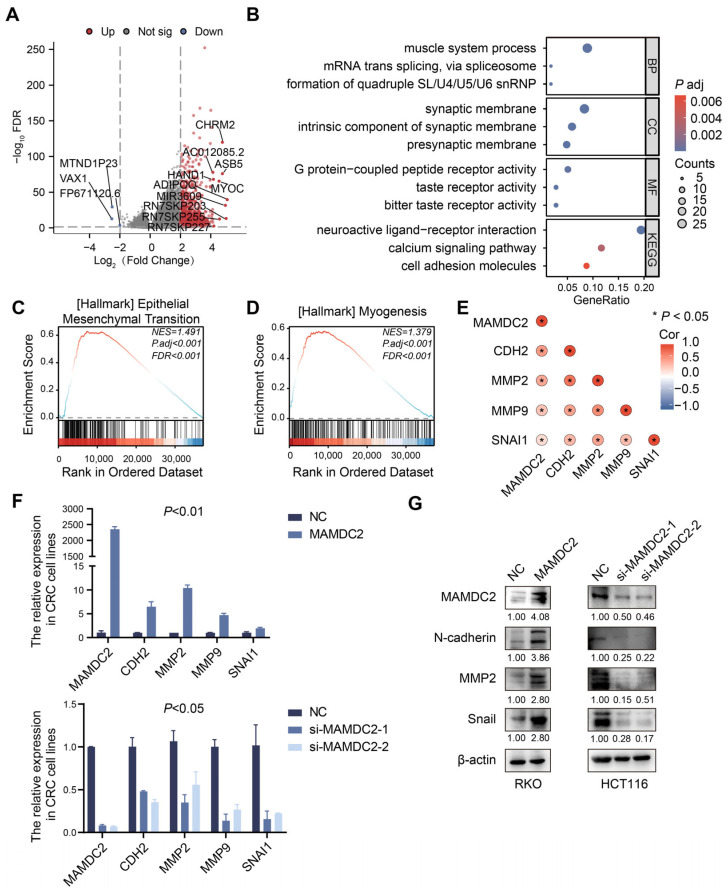
MAMDC2 regulates epithelial–mesenchymal transition in colorectal cancer. (**A**) *MAMDC2* single gene difference analysis. (**B**) GO and KEGG based on *MAMDC2* single gene differential expression. (**C**,**D**) EMT gene set and myogenesis gene set from GSEA based on *MAMDC2* single gene differential expression gene set. (**E**) Correlation between *MAMDC2* and EMT-related genes. (**F**) The real-time RT-PCR analysis of *CDH2*, *MMP2*, *MMP9*, and *SNAI1* with *MAMDC2* overexpression (**above**) or knockdown (**below**). (**G**) Western blot analysis of EMT-related proteins (N-cadherin, MMP2, and Snail) in RKO with MAMDC2 overexpression (**left**) and HCT116 with MAMDC2 knockdown (**right**).

To better understand the potential mechanism by which *MAMDC2* contributes to the development of CRC, we used TCGA-COAD data to perform differentially expressed gene (DEG) analysis. A total of 1901 DEGs were revealed (|LogFC| > 2 and FDR < 0.05), including 1898 upregulated and 3 downregulated genes (Figure 5A). To further identify genes functionally associated with *MAMDC2*, we used the DEGs to conduct GO and KEGG analyses, which showed 223 biological processes (BPs), 49 cellular components (CCs), 41 molecular functions (MFs), and 8 KEGG pathways. Bubble charts showing the top three enriched GO and KEGG pathways suggest that *MAMDC2* may play an important role in tumorigenesis *and* development (Figure 5B). Moreover, we performed GSEA, which demonstrated that *MAMDC2* was significantly enriched in the gene sets associated with EMT (NES = 1.491, *P*.adj < 0.001, FDR < 0.001) and myogenesis (NES = 1.379, *P*.adj < 0.001, FDR < 0.001), suggesting a potential association between *MAMDC2* and CRC metastasis-related pathways (Figure 5C,D). In addition, analysis of the TCGA database revealed that *MAMDC2* was positively correlated with EMT-related genes at the mRNA level, and the correlation coefficient was high (*p* < 0.05) (Figure 5E). These results suggest that *MAMDC2* may be involved in the EMT process and affects this process by regulating the expression of EMT-related genes. To verify this observation, we examined the mRNA expression of *MAMDC2* in CRC cell lines and selected RKO and HCT116 for functional studies (Figure S1A). RKO cells were transfected with MAMDC2 overexpression plasmids, while HCT116 cells were treated with siRNA targeting MAMDC2. The results of real-time RT-PCR showed that MAMDC2 overexpression significantly upregulated the mRNA levels of *CDH2*, *MMP2*, *MMP9*, and *SNAI1*, whereas MAMDC2 knockdown had the opposite effect (Figure 5F and Figure S1B). Consistently, Western blot analysis revealed increased protein expression of N-cadherin, MMP2, and Snail in MAMDC2 overexpression cells, whereas their levels were decreased in MAMDC2 knockdown cells (Figure 5G). Similar trends were observed in LoVo (overexpression) and SW480 (knockdown) cells (Figure S1C). These results indicate that MAMDC2 plays an important role in regulating EMT.

### 3.6. Result 6: Co-Expression Patterns of MAMDC2 and MYLK in Colorectal Cancer

**Figure 6:**
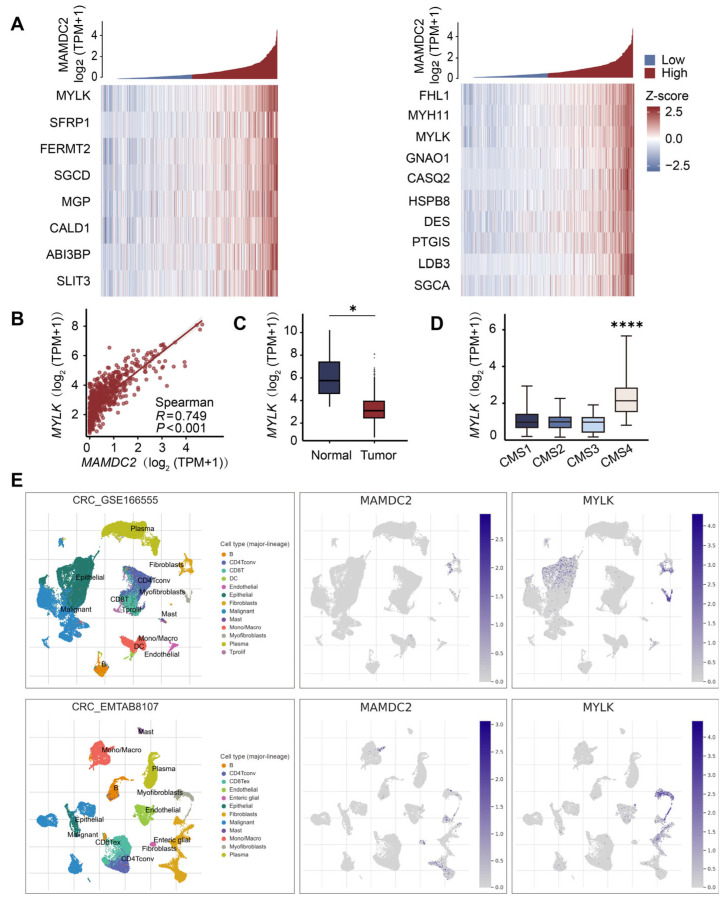
Co-expression patterns of MAMDC2 and MYLK in colorectal cancer. (**A**) Correlation between *MAMDC2* and the top 10 genes in the EMT gene set and myogenesis gene set. (**B**) Correlation between *MAMDC2* and *MYLK*. (**C**) The expression of *MYLK* in CRC tissues. n (normal) = 359. n (tumor) = 383. (**D**) The expression level of *MY*L*K* in different CMS subtypes of CRC. n (CMS1) = 84. n (CMS2) = 145. n (CMS3) = 71. n (CMS4) = 138. (**E**) Localization and expression of *MAMDC2* and *MYLK* in single-cell sequencing data (GSE166555 and EMTAB8107). * *p* < 0.05, **** *p* < 0.0001.

To identify the key genes contributing to the EMT pathway, we conducted a comprehensive co-expression analysis and visualized the top 10 most significant DEGs that are co-expressed between *MAMDC2* and the EMT and myogenesis gene sets (Figure 6A,B). MYLK is a calcium/calmodulin-dependent enzyme that regulates light chains by phosphorylating myosin and promotes the interaction of myosin with actin filaments to produce contractile activity. Next, we used the TCGA-COAD to analyze the mRNA expression levels of the *MYLK* in normal and tumor tissues. The results showed that *MYLK* was expressed at abnormally low levels in CRC tissues compared with normal tissues (*p* < 0.05) (Figure 6C). We further analyzed the expression of *MYLK* in different CMS subtypes. The results showed that *MYLK* was highly expressed specifically in CMS4 CRC (*p* < 0.0001), which was consistent with the expression of *MAMDC2* in CMS4 CRC (Figure 6D). To clarify the potential mode of the interaction network between *MYLK* and *MAMDC2*, we used single-cell sequencing data (GSE166555 and EMTAB8107) to explore the distribution and expression of the two genes in each malignant cell. The results showed that *MYLK* and *MAMDC2* have highly overlapping cell localizations, such as malignant epithelial cells, fibroblasts, and myofibroblasts (Figure 6E). These findings reveal the potential synergistic role of MYLK and MAMDC2 in CRC.

### 3.7. Result 7: Cancer Cell-Derived MAMDC2 Promotes MYLK Expression in CAFs

**Figure 7:**
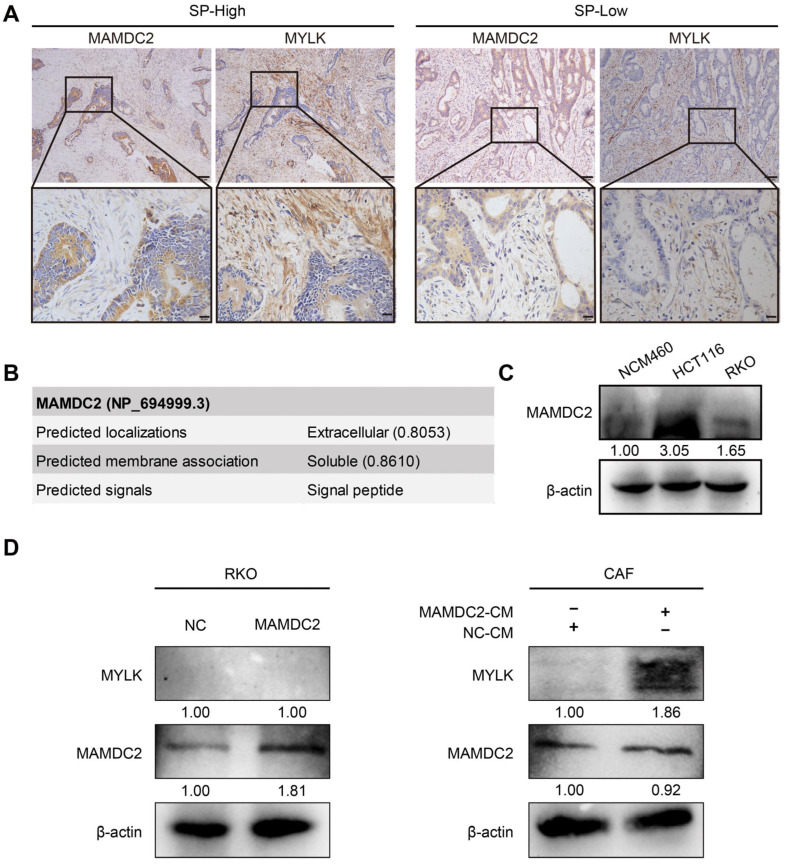
Cancer cell-derived MAMDC2 promotes MYLK expression in CAFs. (**A**) Expression of MAMDC2 and MYLK in CRC with different TSRs. Original magnifications 40× and 100×. n (SP-L) = 85. n (SP-H) = 36. (**B**) Predicted subcellular localization and membrane association of MAMDC2. (**C**) Western blot analysis of MAMDC2 in NCM460, HCT116, and RKO culture supernatant. (**D**) Western blot analysis of MAMDC2 overexpression in RKO and its regulatory effect on MYLK expression in both RKO (**left**) and CAFs (**right**).

Next, we explored the expression characteristics and potential interactions of MYLK and MAMDC2 in CRC. IHC showed that MYLK was located mainly in the cytoplasm of CAFs, and its expression level in high-TSR CRC was significantly higher than that in low-TSR CRC, which was consistent with the expression pattern of MAMDC2, suggesting that the two genes may be co-expressed (Figure 7A). To further characterize MAMDC2, we employed DeepLoc-2.1 for subcellular localization prediction. The analysis indicated that MAMDC2 is most likely localized to the extracellular space (probability: 0.8653) and classified as a soluble protein (probability: 0.8608). Notably, MAMDC2 was predicted to contain a signal peptide sequence, reinforcing its identity as a secreted protein (Figure 7B). Consistent with this, Western blot detected abundant MAMDC2 in the culture supernatant, confirming that it is secreted into the extracellular space (Figure 7C). To further verify this hypothesis, we used Western blotting and found that the overexpression of MAMDC2 alone did not directly affect the expression level of MYLK in RKO cells. However, when RKO cells overexpressing MAMDC2 were cocultured with CAFs for 48 h, the expression of MYLK in CAFs increased significantly, indicating that the high expression of MAMDC2 in CRC cells may induce the upregulation of MYLK in CAFs through paracrine mechanisms (Figure 7D), and a similar trend was observed in HCT116 (Figure S2). Based on these results, we speculate that MAMDC2, as a secreted protein, may be released from tumor cells into the tumor microenvironment, where it regulates MYLK expression in CAFs. This mechanism might contribute to CAF activation and proliferation, consequently promoting CRC invasion and progression.

## 4. Discussion

In the consensus molecular classification of CRC, the CMS4 subtype accounts for approximately 23% of cases. This subtype is characterized by high invasiveness, strong metastatic potential, rapid tumor progression, and a significantly increased risk of peritoneal metastasis [2]. In addition, CMS4 CRC has a poor response to conventional chemotherapy and suboptimal outcomes with immunotherapy, resulting in unfavorable five-year and relapse survival rates. Therefore, early diagnosis of CMS4 CRC and implementation of personalized, multidisciplinary treatment strategies are critical to improve patient prognosis. However, CMS4 is typically associated with high chromosomal instability (CIN), low mutation count, and high copy number, necessitating reliance on gene sequencing for diagnosis [2]. This diagnostic process is not only time-consuming; it also involves significant technical costs, which place an additional financial burden on patients. Therefore, there is an urgent clinical need to identify effective and easily detectable diagnostic biomarkers.

Recent advances in CRC research have broadened the focus from tumor cells themselves to broader aspects of the tumor microenvironment and non-cellular components. MAM domain-containing proteins are typically involved in biological processes such as cell adhesion, signal transduction, and maintenance of cell polarity [14]. This study is the first to investigate the expression profiles of 18 MAM domain-containing molecules in CRC tissue samples from The Cancer Genome Atlas (TCGA). Through bioinformatic analysis, we identified four MAM domain-containing genes (*EGFL6*, *MEP1A*, *MEP1B*, and *MAMDC2*) that showed significant differential expression between tumor and adjacent normal tissues in CRC. Of these, *EGFL6* encodes a member of the epidermal growth factor repeat superfamily, characterized by EGF-like repeats, and is a pro-angiogenic factor specifically secreted by osteoblasts that regulates bone vascularization [29]. MEP1A is predicted to have metalloendopeptidase activity, is involved in proteolytic processes, is localized in extracellular vesicles, and promotes the progression of hepatocellular carcinoma [30]. Meprin β (encoded by *MEP1B*) is a type I transmembrane protein that functions as a zinc-dependent metalloendopeptidase. Structurally, it comprises three functional domains: the MAM domain, metalloprotease catalytic domain, and TRAF domain. The MAM domain is particularly critical for both membrane localization and the “sheddase” activity of meprin β, which mediates ectodomain shedding of membrane-anchored proteins. Notably, meprin β specifically cleaves E-cadherin. Loss of meprin β function has been strongly associated with tumor progression, suggesting its essential role in maintaining cellular adhesion and suppressing tumorigenesis [31,32,33]. Although *EGFL6* is highly expressed in CMS4 CRC, it lacks CMS4-specific expression patterns. In contrast, *MAMDC2* is significantly enriched in CMS4 and shows significant differences compared to CMS1-3, suggesting that MAMDC2 may play an important role in CMS4 CRC.

MAMDC2 is a protein containing four MAM domains. Pan-cancer analysis shows that *MAMDC2* is significantly downregulated in 15 malignancies, including urothelial carcinoma, invasive breast cancer, cervical cancer, and endometrial cancer. Current research on MAMDC2 expression in tumors is primarily limited to the transcript level, with a lack of comprehensive protein expression data, particularly in CRC, where systematic analysis of MAMDC2 protein expression remains unexplored.

Diagnosis of CMS4 CRC relies on gene sequencing, which limits the availability of tissue samples [34,35]. Long-term clinical studies suggest that MSS-type CRC with high stromal content shares significant similarities with CMS4 CRC in terms of gene expression alterations, tumor cell behavior, and clinical prognosis. Based on this, we performed an immunohistochemical analysis on 121 CRC tissue samples collected between 2017 and 2023 and found that MAMDC2 expression is significantly higher in high-TSR CRC tissues compared to low-TSR tissues. In addition, MAMDC2 expression is significantly increased in peritoneal metastatic tissues but decreased in liver metastatic tissues. Reports in the literature suggest that CMS subtypes in peritoneal metastases are relatively homogeneous, predominantly CMS4, whereas CMS subtype switching is more common in liver metastases, with CMS2 accounting for approximately 60% of cases [36]. This is consistent with our previous findings. In conclusion, we have identified MAMDC2 as a highly expressed molecule in MSS CRC with a high TSR, suggesting its potential role as a stromal-associated effector. Notably, MAMDC2 is significantly enriched in highly stromal peritoneal metastatic tissues, with its expression levels positively correlating with tumor stromal abundance, likely induced by the tumor stromal microenvironment and driving CRC progression through tumor stroma interactions.

To further elucidate the functional mechanisms of MAMDC2, we performed KEGG and GO enrichment analyses. The results indicate that *MAMDC2* is significantly enriched in gene sets related to EMT and myogenesis, suggesting its potential role in regulating CRC invasion, metastasis, and stromal remodeling. EMT is a critical biological process in tumor progression and metastasis that allows epithelial cells to acquire mesenchymal phenotypes, thereby enhancing their migratory and invasive capabilities. In CRC, EMT is closely associated with distant metastasis and is involved in the remodeling of the tumor microenvironment through mechanotransduction-related signaling pathways [37,38]. Studies have shown that mechanical signals, such as extracellular matrix stiffness and tension, can activate EMT-related transcription factors (e.g., SNAIL, TWIST, and ZEB1) to drive invasive tumor phenotypes [39,40]. In addition, mechanical signals promote collective tumor cell migration and metastatic niche formation by regulating cytoskeletal reorganization and cell matrix interactions [41,42]. The co-enrichment of *MAMDC2* in EMT and myogenesis-related pathways implies a potential association between MAMDC2 and CRC invasion and metastasis, possibly mediated by mechanical signaling or EMT-related processes. However, whether MAMDC2 directly activates EMT transcription factors or modulates extracellular matrix stiffness requires further experimental validation.

Although evidence suggests that MAMDC2 plays a tumor-suppressing role in ER+ breast cancer, our study found that MAMDC2 is significantly upregulated in CMS4 CRC and correlates with aggressive tumor features, such as high TSR and peritoneal metastasis, and promotes the EMT of CRC cells, suggesting it may have a tumor-promoting effect. This functional difference may stem from specific cellular environments and tumor microenvironments. For instance, the tumor microenvironment in CMS4 CRC is characterized by abundant desmoplastic stroma and activated CAFs, which may alter the functional output of MAMDC2 through paracrine signaling or microenvironmental remodeling. Furthermore, gene expression regulation and downstream signaling pathways of MAMDC2 could differ between these cancers.

Further exploration of the functional mechanisms of MAMDC2 identified MYLK as a key molecule in EMT and myogenesis pathways through functional annotation and pathway analysis. *MYLK* (myosin light-chain kinase) is a key marker gene for myofibroblasts identified with single-cell transcriptome analysis and is primarily involved in the regulation of cytoskeletal reorganization, cell contraction, and mechanotransduction [43,44,45]. In tumors, MYLK promotes cell migration, invasion, and stromal remodeling, playing an important role in tumor progression and metastasis [46,47,48]. Studies have shown that 12(S)-HETE, a lipid mediator, activates MYLK through the Ca^2+^/RHO/ROCK signaling axis, leading to the phosphorylation of MLC2 (myosin light-chain 2). This process induces cell contraction and the formation of cancer cell-induced pores (CCIDs), facilitating cancer cell invasion. In CAFs, 12(S)-HETE-induced MYLK activation enhances CAF contractility and motility, creating favorable conditions for tumor cell invasion [49]. Our results show a significant positive correlation between MAMDC2 and MYLK, suggesting a potential functional association. Furthermore, MYLK expression and localization closely resemble those of MAMDC2, indicating possible mechanistic links. Coculture experiments further confirmed that overexpression of MAMDC2 in CRC cells upregulates MYLK in CAFs, suggesting that MAMDC2, as a secreted protein, may regulate MYLK expression in CAFs through paracrine mechanisms. However, whether this correlation reflects a direct regulatory relationship or parallel activation by upstream factors (such as TGF-β or mechanical stress) remains to be determined.

Our study has several limitations. First, we preliminarily explored the expression characteristics of MAMDC2 in colorectal cancer and its potential association with the EMT process and MYLK expression in CAFs through bioinformatics analysis and in vitro experiments. However, the specific biological function of MAMDC2 in colorectal cancer remains unclear, and there is currently a lack of in vivo experimental verification. Second, we only verified that MAMDC2 can be secreted by colorectal cancer cells but did not analyze its expression and secretion in CAFs, which is critical to clarify its mode of action in tumor stroma crosstalk. Third, while MAMDC2 shows CMS4-specific expression, its diagnostic sensitivity or therapeutic advantage over CMS4 biomarkers (e.g., TGF-β) remains unclear. In addition, we have not elucidated the precise pathway of tumor-derived MAMDC2 entry into CAFs, the molecular mechanism by which MAMDC2 regulates MYLK expression in CAFs, or its biological effects on CAFs—key questions that require further investigation.

In conclusion, our study is the first to systematically elucidate the expression patterns and functional roles of MAMDC2 in CRC, particularly in MSS colorectal cancer with a high TSR. Our findings suggest that the MAMDC2/MYLK axis may play an important role in tumor stroma interactions and the progression of high-stromal CRC. These results provide new insights into the molecular mechanisms underlying CMS4 CRC and may lay a theoretical foundation for the development of targeted diagnostics. However, further in vivo and mechanistic studies are needed to validate these observations and fully clarify the clinical utility of MAMDC2.

## Data Availability

The datasets utilized in this study are publicly accessible from the following repositories: GSE245552, GSE183916, EMTAB8107, GSE166555, and TCGA.

## Supplementary Materials

The following supporting information can be downloaded at: https://www.mdpi.com/article/10.3390/biomedicines13051217/s1, Figure S1: MAMDC2 regulates epithelial–mesenchymal transition in colorectal cancer. Figure S2: Cancer cell-derived MAMDC2 promotes MYLK expression in CAFs. Figure S3: The western blot original images.

## Abbreviations

The following abbreviations are used in this manuscript:

CRC	colorectal cancer
MAM	meprin/A-5 protein/receptor protein-tyrosine phosphatase mu
TSR	tumor stroma ratio
CMS	consensus molecular subtype
CMS4	The mesenchymal subtype
EMT	epithelial–mesenchymal transition
CAFs	cancer-associated fibroblasts
MAMDC2	MAM domain-containing 2
MYLK	myosin light-chain kinase
LM	liver metastases
PM	peritoneal metastases
DEG	differentially expressed gene
CIN	chromosomal instability
MSS	microsatellite stable stability
