# Supplementary material for: The Expression and Molecular Roles of MAMDC2 in MSS Colorectal Cancer with a High Tumor Stromal Ratio
Source: Biomedicines. 2025 May 17;13(5):1217. doi: 10.3390/biomedicines13051217 (PMC12109205; doi:10.3390/biomedicines13051217)
Supplement: Supplementary file 1 [file biomedicines-13-01217-s001.zip › Figure S1.MAMDC2 regulates epithelial–mesenchymal transition in colorectal cancer.pdf]

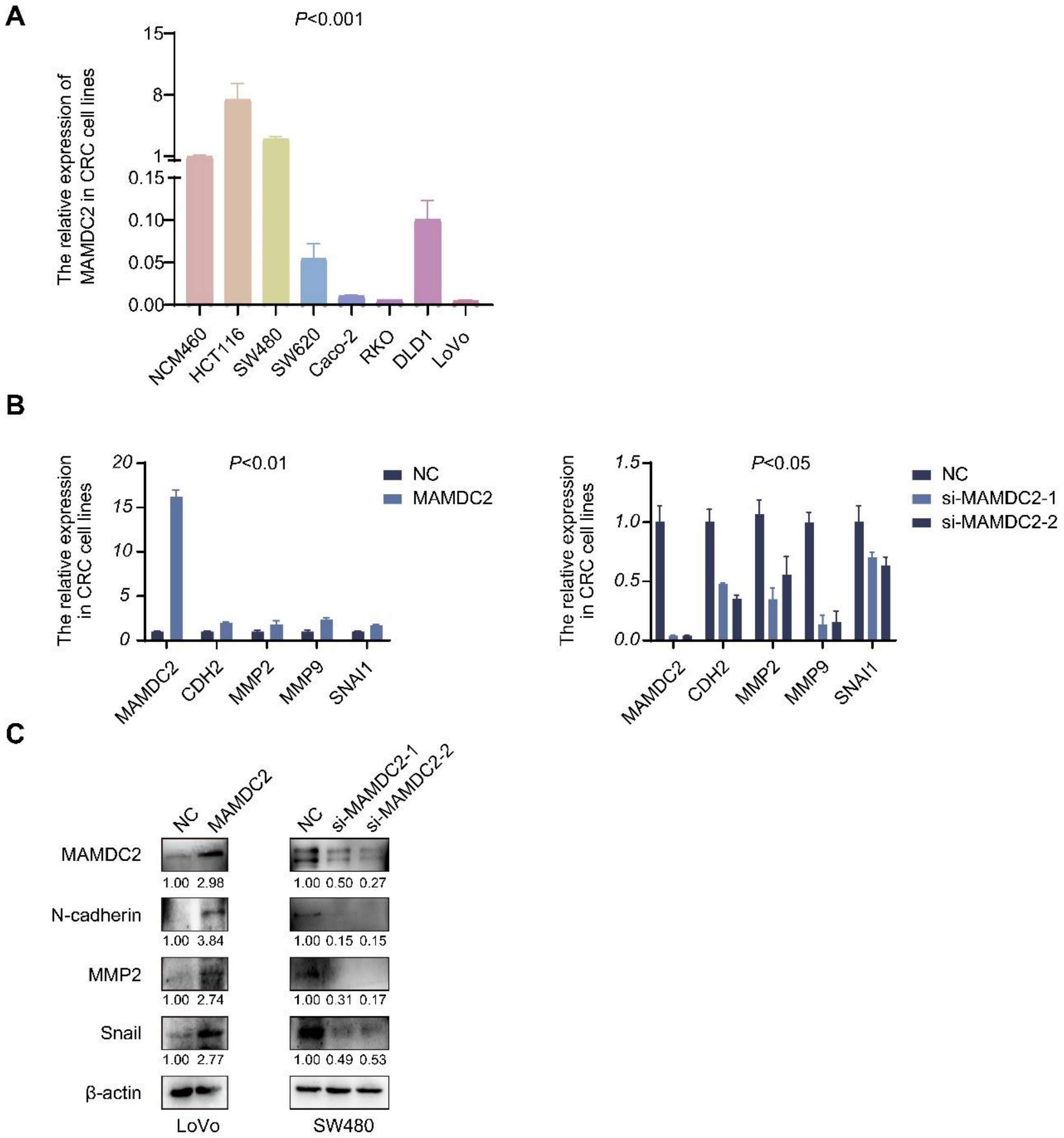

Figure S1. A. The relative expression of MAMDC2 in CRC lines. B. The RT- PCR analysis of *CDH2, MMP2*, *MMP9*, and *SNAI1* in LoVo with MAMDC2 overexpression (left) and SW480 with MAMDC2 knockdown (right). C. Western blot analysis of EMT-related proteins (N-cadherin, MMP2, and Snail) in LoVo with MAMDC2 overexpression (left) and SW480 with MAMDC2 knockdown (right).
