# Supplementary material for: The Expression and Molecular Roles of MAMDC2 in MSS Colorectal Cancer with a High Tumor Stromal Ratio
Source: Biomedicines. 2025 May 17;13(5):1217. doi: 10.3390/biomedicines13051217 (PMC12109205; doi:10.3390/biomedicines13051217)
Supplement: Supplementary file 1 [file biomedicines-13-01217-s001.zip › Figure S2.Cancer cell-derived MAMDC2 promotes MYLK expression in CAFs..pdf]

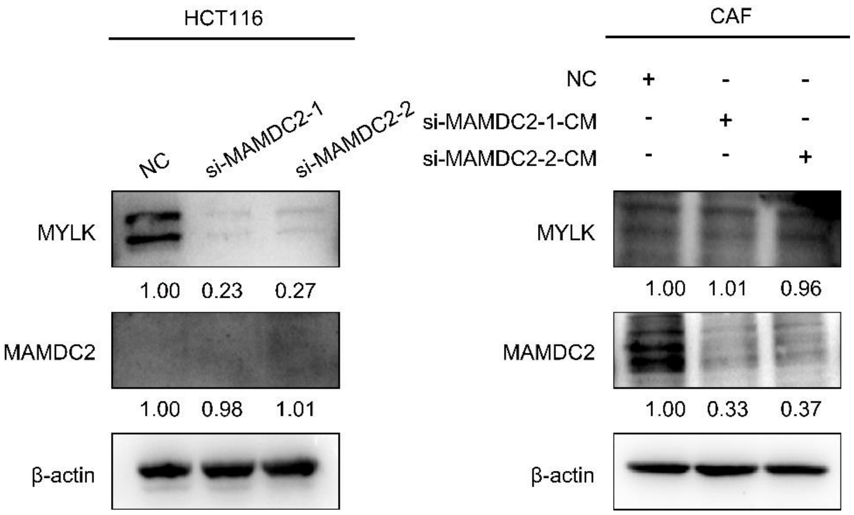

Figure S2. Western blot analysis of MAMDC2 overexpression in HCT116 and its regulatory effect on MYLK expression in both HCT116 (left) and CAFs (right).
