# Supplementary figures and images for: The Expression and Molecular Roles of MAMDC2 in MSS Colorectal Cancer with a High Tumor Stromal Ratio
Source: Biomedicines. 2025 May 17;13(5):1217. doi: 10.3390/biomedicines13051217 (PMC12109205; doi:10.3390/biomedicines13051217)

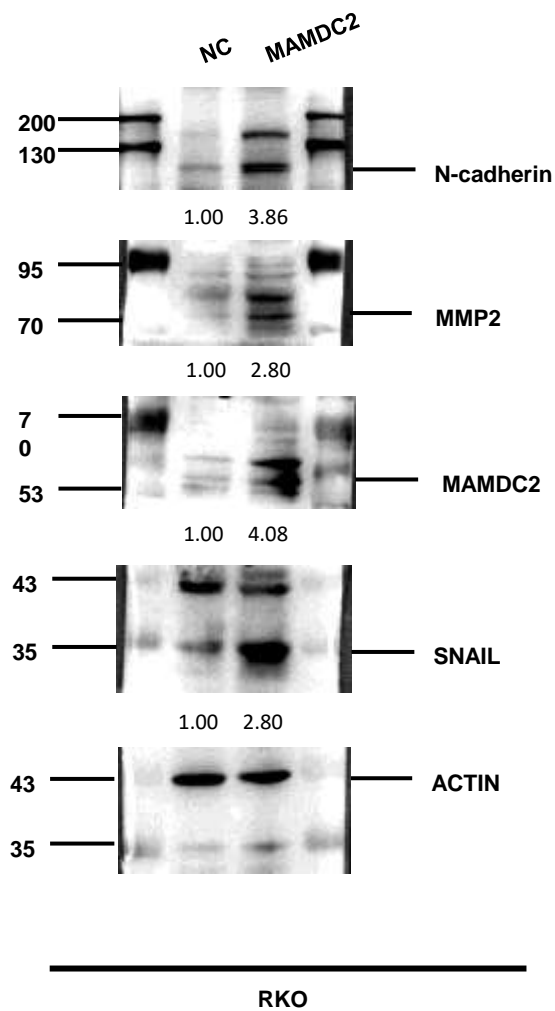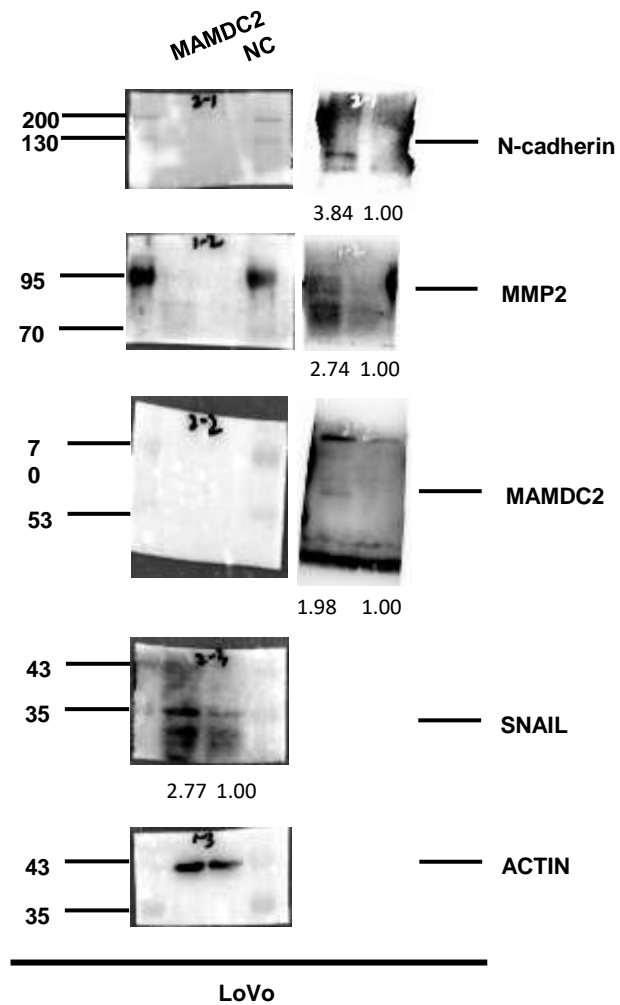

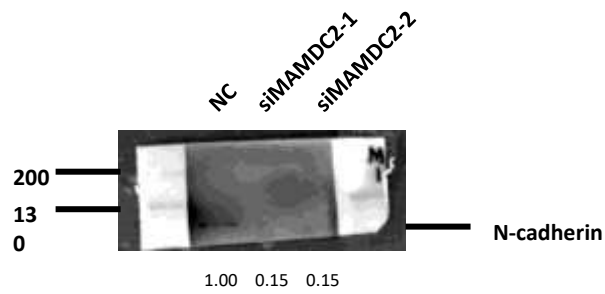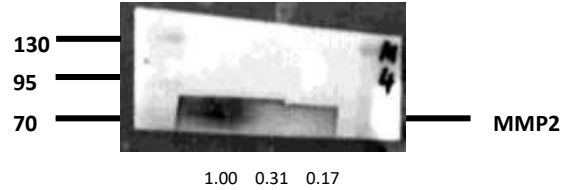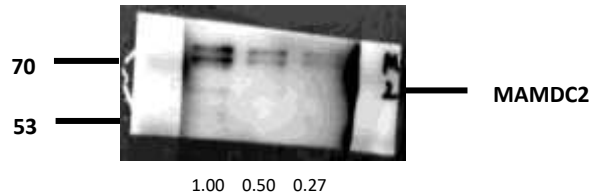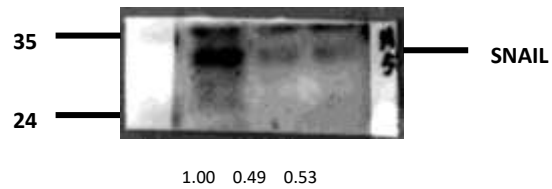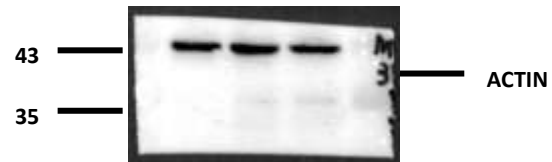

SW480

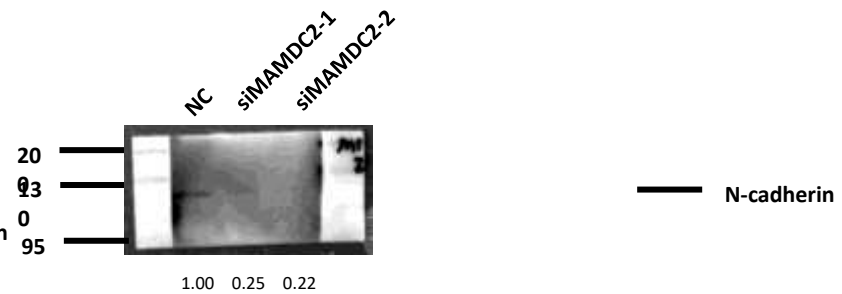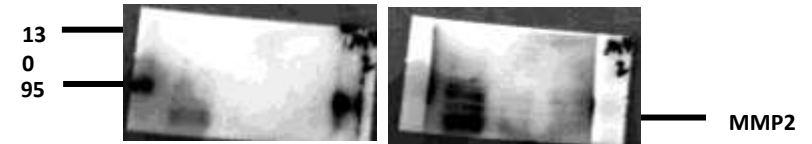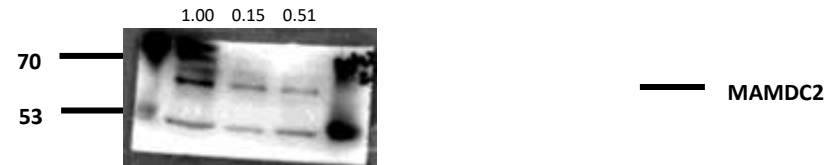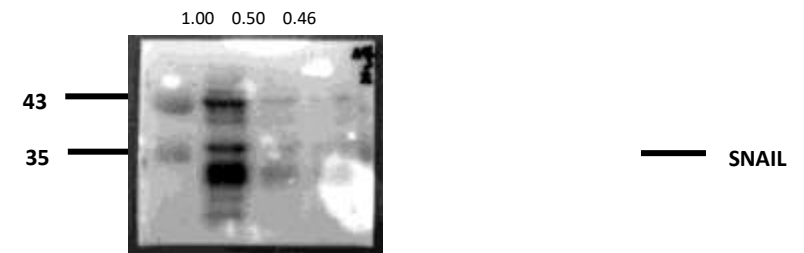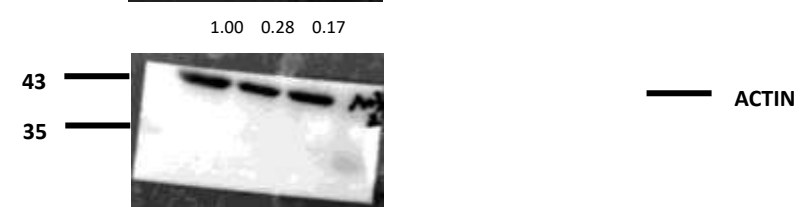

HCT116

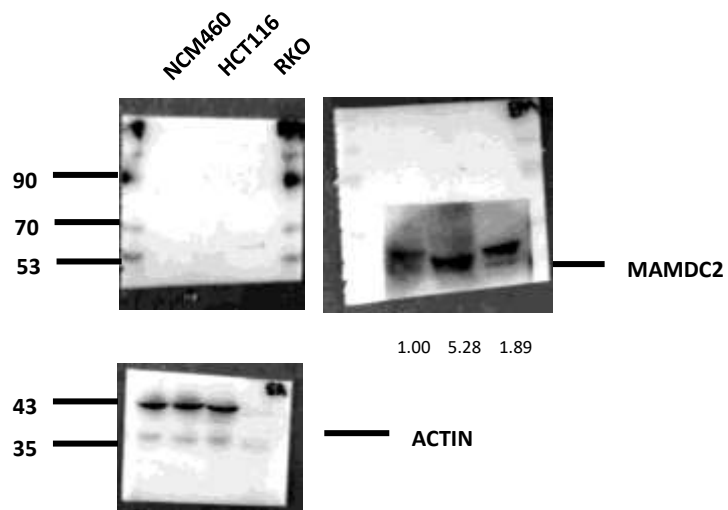

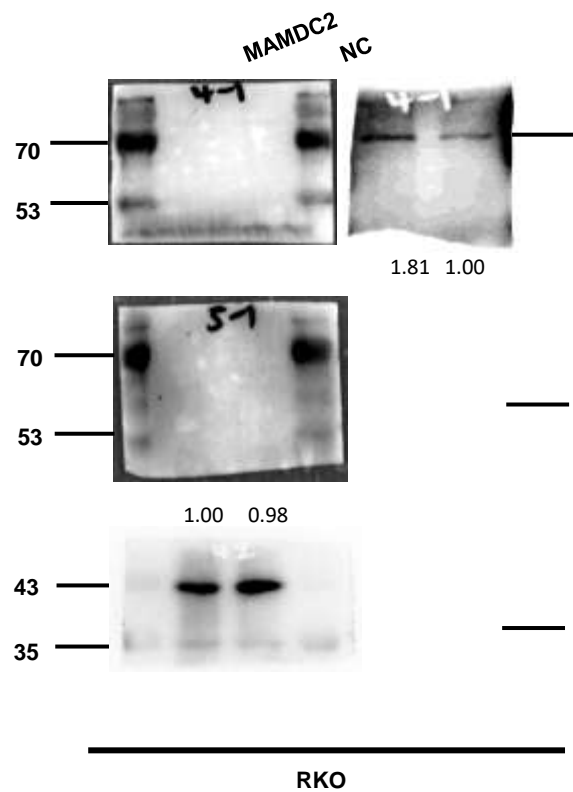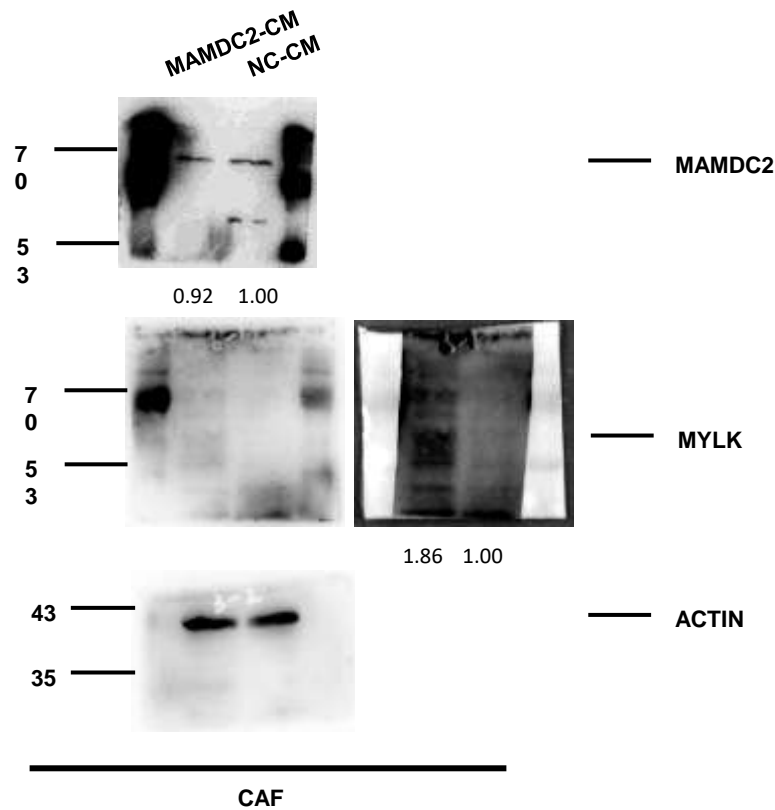

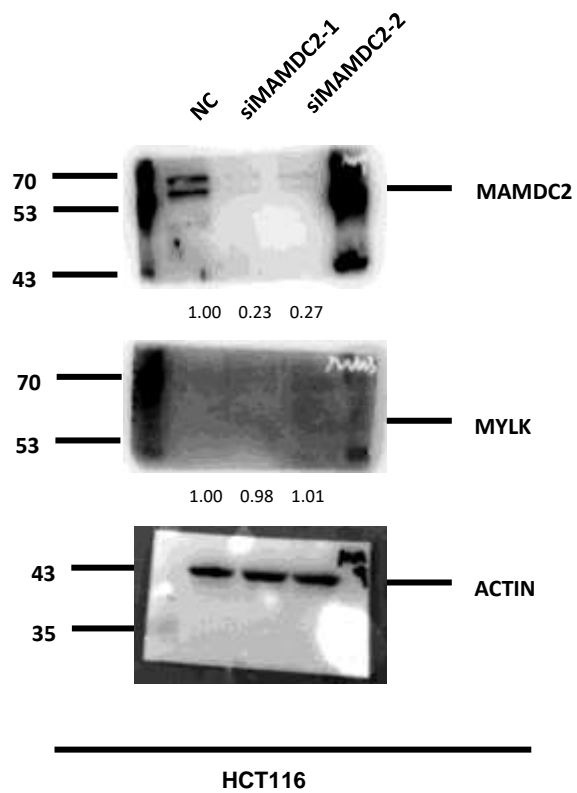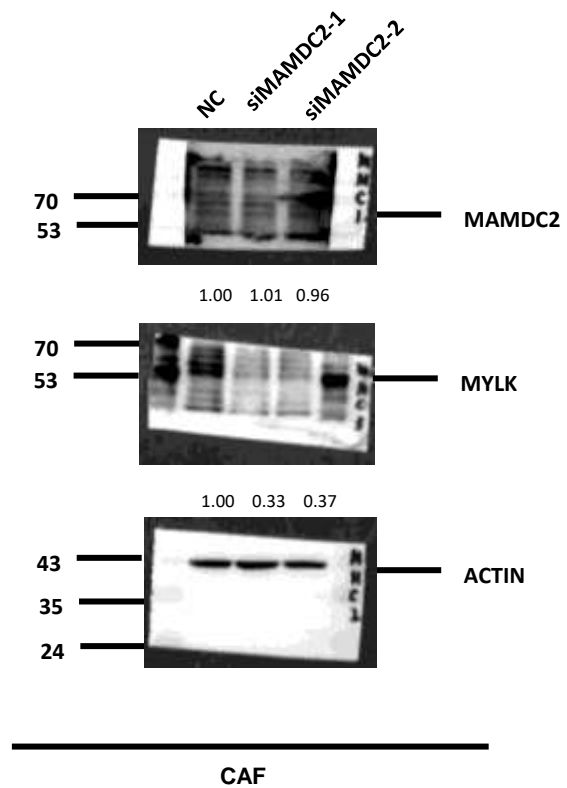

Supplement: Supplementary file 1 [file biomedicines-13-01217-s001.zip › Figure S3.The western blot original images.pdf]
